# Enhanced Recovery in Surgical Intensive Care: A Review

**DOI:** 10.3389/fmed.2018.00256

**Published:** 2018-10-04

**Authors:** Gordana Jovanović, Dea Karaba Jakovljević, Mirka Lukić-Šarkanović

**Affiliations:** ^1^University of Novi Sad, Faculty of Medicine, Department of Anesthesia and Perioperative Medicine, Novi Sad, Serbia; ^2^Clinic for Anesthesia and Intensive Care Therapy, Clinical Center of Vojvodina, Novi Sad, Serbia; ^3^University of Novi Sad, Faculty of Medicine, Department of Physiology and Sports Medicine, Novi Sad, Serbia

**Keywords:** ERAS protocol, surgical intensive care unit, recovery, perioperative care, early mobilization

## Abstract

Patients are admitted to the surgical intensive care (SICU) unit after emergency and elective surgery. After elective surgery, for further support, or to manage coexisting comorbidities. The implementation of the ERAS (Enhanced recovery after surgery) protocols in surgery should decrease the need for ICU beds, but there will always be unpredicted complications after surgery. These will require individual management. What we can do for our surgical patients in ICU to further enhance their recovery? To promote early enhanced recovery in surgical intensive care—SICU, three areas need to be addressed, sedation, analgesia, and delirium. Tools for measurement and protocols for management in these three areas should be developed to ensure best practice in each SICU. The fourth important area is Nutrition. Preoperative screening and post-operative measurement of the state of nutrition also need to be developed in the SICU. The fifth important area is early mobilization. ERAS protocols encourage early mobilization of the critically ill patients, even if on mechanical ventilation. Early mobilization is possible and should be implemented by special multidisciplinary ICU team. All team members must be familiar with protocols to be able to implement them in their field of expertise. Personal and professional attitudes are critical for implementation. In the core of all our efforts should be the patient and his well-being.

## Introduction

### History of enhanced recovery concept

Enhanced recovery concept was first introduced by Henrik Kehlet in 1990's. As a colorectal surgeon he developed the idea of “fast tracking” in major abdominal surgery, in order to promote faster recovery, and diminish complication after surgery.

Factors that limit early recovery include pain, post-operative paralytic ileus and different organ system dysfunctions. The traditional approach in use for decades, utilize only unimodal solutions for these problems (1–3).

For enhanced recovery, a multimodal, evidence - based approach is proposed.

Implementation of this innovative concept was facilitated by the simultaneous development of new regional anesthesia techniques for pain control, and minimally invasive laparoscopic techniques in abdominal surgery.

In 2010, ERAS (Enhanced recovery after surgery) Society was formed as a nonprofit, academic, international multidisciplinary society and has played an important role in helping to provide guidelines, educational meetings, and other support (www.erassociety.org) (2–4). The mission of the ERAS Society is to develop peri-operative care and improve recovery through research, education, audit and implementation of evidence-based practice.

To the present day 9 ERAS Society guidelines are available: for colonic surgery, pancreaticoduodenestomy, elective rectal/pelvic surgery, radical cystectomy, liver, bariatric, head and neck cancer surgery, gastrectomy and breast surgery (3). Modified ERAS guidelines exist for gynecology, thoracic, vascular, pediatric, urologic, orthopedic, esophagectomy, and colorectal liver metastasis surgery.

As authors, we are not aware of the existence of ERAS protocols specific for surgical intensive care, and although all existing ERAS protocols have an influence on patients being admitted in surgical intensive care unit, we think that more can be done, to further enhance recovery of these patients.

## Early recovery protocols as concept in surgical intensive care

Patients are admitted to the intensive care unit after emergency or elective surgery. In case of admission after elective surgery, for further support to manage coexisting comorbidities.

ERAS protocols in surgery starts in the preoperative period, and potential need for post-operative ICU admission should be discussed with the patient preoperatively.

### Surgical intensive care unit

As JL Vincent highlighted, a neglect of recovery is one of the ten big mistakes in intensive care medicine (5). Everyday battle for patient survival draws attention from recovery and period after leaving the intensive care unit.

In theory, the implementation of the ERAS protocols in surgery should decrease the need for ICU beds, but will always be unpredicted complications after surgery, which will be managed individually.

The next question is what can be done for our surgical patients in SICU to enhance their recovery?

### The role of sedation and analgesia in enhanced recovery after surgery in the intensive care unit

Sedation and analgesia are an integral part of care for critically ill, but sometimes with low priority, as care for other organ systems, as cardiac, pulmonary, or renal system are more acutely in focus. Only a small percentage of SICU patients need prolonged deep sedation. In the most cases the objective is for the patient to remain calm, cooperative and comfortable. The concept of “e CASH” (**e**arly **C**omfort using **A**nalgesia, minimal **S**edatives and maximal **H**uman care) is being introduced to guide clinicians (5–7).

Using this concept strategies for sedation and analgesia in SICU can be provided.

#### Analgesia

All critically ill adult patients in medical, surgical, and trauma ICUs typically experience pain. Providing analgesia should be a priority, on humanitarian grounds, and in keeping with the ERAS concept.

Essential for early mobilization, multimodal approach to pain should be implemented (8).

As stated in recent guidelines “Multimodal analgesia or balanced analgesia is approach that involves the use of more than one method or modality of controlling pain. These modalities may operate through different mechanisms or at different sites (i.e., peripheral vs. central actions). One example of multimodal analgesia is the use of various combinations of opioids and local anesthetics to manage post-operative pain” (9).

Pain management is the mainstay of treatment and precondition for other treatment options such as early mobilization where multimodal approach to pain should be implemented (8).

Pain should be measured in order to regularly reevaluate and optimize treatment.

There are many tools for measuring the pain in critically ill patient (Critical Car**e Pain Observation Tool or BPS**—Behavioral Pain Scale) which can be used, or modified for local conditions (10).

#### Sedation and delirium

Sedation is integral part of care in SICU to relieve discomfort and unnecessary suffering. Over sedation on the other hand is not beneficial and only a small percentage of patient need profound sedation and immobilization (5, 6). Benzodiazepines in the sedation of SICU patients should be avoided, and non-sedative drugs use such as propofol or dexmedetomidine should be encouraged (8).

Delirium is a common problem in some SICU patients affecting outcome in terms of prolonged ICU stay, persistent cognitive decline, reintubation, and worse overall outcome. Delirium is frequent in the medical and surgical Intensive Care Units (ICUs) with prevalence rates ranging from 32.3 to 77% and the incidence rates varying from 45 to 87% (11–14).

Currently there is a study recruiting patients “UNDERPIN-ICU” consisting of standardized protocols focusing on several modifiable risk factors for delirium, including cognitive impairment, sleep deprivation, immobility and visual and hearing impairment. The future results and maybe some implementation of the protocol remains to be seen (15).

In preventing delirium in ICU, there is a great potential in involving family members in activities that constantly serve as an orientation, cognitive or sensitive stimulus to the patients (16).

#### Patient-centered care

The patient should be in the center of all our efforts. Patient well-being and needs are our priority. Counseling, emotional support, and humanistic approach should be implemented in our everyday work. Many non-pharmacological modalities can be employed in sleep promotion and as alternative means of communication (5–7).

There are recent Clinical practice guidelines for the management of pain, agitation, and delirium in adult patients in the intensive care unit (8), that should be used according to local organization of the health care system.

The 2013 PAD guidelines (**P**ain **A**gitation **D**elirium) have been incorporated into the ICU PAD Care Bundle, with corresponding metrics developed to facilitate implementation. The bundle emphasizes an integrated approach to assessing, treating and preventing significant pain, over or under sedation, and delirium in critically ill patients (8, 12).

To promote early enhanced recovery in SICU, we should provide protocols for sedation and analgesia in the SICU (or develop local ones), implement tools for assessing the patient level of sedation and pain, develop and implement prevention and treatment strategies for post-operative delirium. The best practice will be to develop and implement care bundles for sedation, analgesia and post-operative delirium in each SICU.

## The role of nutrition in enhanced recovery after surgery in the intensive care unit

### Early nutrition in SICU

Patients undergoing surgery are subjected to a catabolic state through activation of the inflammatory response and the release of stress hormones—cortisol, catecholamine, and glucagon. The stress hormones and inflammatory cytokines exert catabolic effects by depleting liver glycogen stores and causing lipolysis and proteolysis of fat and muscle protein stores. Additionally, surgery naturally induces a state of insulin resistance or decreased insulin sensitivity, with breakdown of lean body mass. This has been associated with increased morbidity, increased mortality, and increased length of hospital stay (17, 18).

Providing nutrition and correcting existing nutritional deficiencies is one of the mainstays in all surgical ERAS protocols. Nutrition support in SICU is also of high importance and interconnected with other elements of care. Nutritional support provides optimal wound healing and prevent loss of lean body mass. Prolonged period of fasting causes breakdown of the gastrointestinal tract barrier function, atrophy of endothelial *microvilli* and decrease of gut lymphoid tissue. This process increases infection and development of sepsis (17, 18).

At the present moment, there are two guidelines: one from ESPEN (European Society for Enteral and Parenteral Nutrition) and one from A.S.P.E.N (American Society for Enteral and Parenteral Nutrition). Both guidelines support the idea of early start with enteral nutrition in critically ill patients. The only contraindications for early enteral nutrition are the conditions of uncontrolled shock and high doses of vasoactive drugs, life threatening hypoxia, hypercapnia, acidosis, or bowel ischemia (19, 20).

The most recent guidelines developed by ESICM (European Society of Intensive Care Medicine) Working group on Gastrointestinal Function within the Metabolism, Endocrinology and Nutrition made 23 recommendations for early enteral nutrition in critically ill patient. Early enteral nutrition is recommended in cases of concomitant usage of neuromuscular blockade, therapeutic hypothermia, extracorporeal membrane oxygenation, prone positioning, for patients with traumatic brain injury, patients with stroke, spinal cord injury, severe acute pancreatitis, after gastrointestinal surgery, after abdominal aortic surgery, abdominal trauma after confirmed/restored gastrointestinal tract, diarrhea (21).

Apart from above mentioned contraindications, delayed enteral nutrition is considered in cases of high output intestinal fistulas, progressing intraabdominal hypertension, abdominal compartment syndrome, acute upper gastrointestinal bleeding, and life threatening metabolic derangements (20, 21).

### The amount of protein in critically ill

The exact amount of protein required in critically ill patient is still a matter of the debate among nutritionists involved in ICU. For the first 12 days in the ICU, an average ICU patient worldwide receives 1,034 kcal/day and 0.6 g/kg/protein (22).

After the first phase of the critical illness (“ebb phase”) the patient enters the recovery phase, total protein, and calorie delivery needs to increase significantly. The total recommended protein intake of 1,2 g/kg/BW /day should increase to 1.5–2 g/kg/BW/day (23, 24).

There is scientific evidence that adequate protein intake leads to better patient functional recovery and quality of life after ICU discharge (22–24).

To enhance further recovery of surgical critically ill patient, implementation of existing protocols should be a high priority. As for pain, agitation, and delirium, screening (measuring) for malnutrition is also very important, and should start preoperatively. Care bundles for early nutritional interventions and implementation of nutritional protocols should be developed by multidisciplinary ICU teams.

## The role of early mobilization in enhanced recovery after surgery in the intensive care unit

Bed rest was first introduced as a medical treatment in the Nineteenth-century to promote recovery, and remains unchanged even today in many hospitals. Critically ill patients are often seen as too sick to be involved in some form of rehabilitation treatment. The rehab programs are frequently perceived as potentially harmful and could cause injury (24).

Immobilization has many deleterious effects on a critically ill patient significantly on the musculoskeletal, cardiovascular, respiratory, and cognitive systems (25, 26).

The development of intensive care unit-acquired weakness (ICUAW) is a longstanding problem in treatment of the critically ill patients and associated with a prolonged duration of mechanical ventilation, delayed rehabilitation, protracted ICU stay, and an increased risk for morbidity and mortality. The cause of ICUAW is multifactorial. There is no specific targeted treatment for prevention of ICUAW. Immobility and bed rest contribute to ICUAW and should be stopped as early as possible (27–29).

Mobilization and physiotherapy for mechanically ventilated patients is an even bigger challenge for health care workers in SICU. Only a small percentage of mechanically ventilated patients are regularly mobilized (<9%) (30, 31).

Prolonged (unnecessary) immobilization during hospitalization in SICU is still a frequent issue. There is mounting evidence that early physical activity and early mobilization are safe and effective. Despite this evidence implementation of this practice is still too low. All efforts aimed to promote this practice of early mobilization and rehabilitation will promote enhanced recovery in those patients (32–34).

ERAS protocols encourage early mobilization of the patients. Early mobilization is possible and should be implemented by special multidisciplinary ICU team.

## Challenges in the implementation of enhanced recovery after surgery in the intensive care unit

It has been more than 15 years from implementation of the first ERAS protocols and global and widespread implementation is yet to occur. It differs greatly among hospitals in different regions of the globe. There are many problems divided into two major categories: one category are caregivers and patients, and the other category are logistic issues (34).

### Health care providers and patients

#### The health care providers

Implementing ERAS concept is more than making protocols. As mentioned above, there are vast scientific data from already existing ERAS protocols. Implementing them requires a multidisciplinary team of surgeons, anesthesiologists, nurses and other team members (nutritionist, physiotherapist, etc.). All team members must be familiar with protocols in order to implement them in their field of expertise. Personal and professional attitudes are critical for implementation. Sometimes enhanced recovery pathways may seem counterintuitive and contradict previous knowledge. For example, early nutrition and early mobilization, because bed rest and nil by mouth was the cornerstone of patient care for decades. All departments need some dedicated physician to promote the idea of enhanced recovery, but sometimes there is a “power play” among different professions over which profession should lead the program (35–37).

#### The patient

As stated previously, in the core of our efforts should be the patient and his well-being.

Effective preoperative counseling is associated with improvements in post-operative recovery in terms of anxiety, faster return of bowel function and improved analgesia. Preoperative counseling is a very important component of the enhanced recovery protocol and should be done at all times. A realistic expectation and proper data are associated with more beneficial health outcomes. The informed patient is capable of making better decisions about their treatment (35, 37).

### Logistic issues

#### Organizational issues

Analyzing enhanced recovery protocols, we come to the conclusion that some of them have more than 20 items.

Implementing all of them will require an organizational modifications in the preoperative anesthesia visit, preoperative preparation, intraoperative pain management or fluid management, modifications in post-operative care, which sometimes results in increased workload, it's time consuming or need additional training (37, 38).

#### Environmental issues

Hospital environment has a positive impact on patient welfare. Specialized ICU, like geriatric ICU with a special features adjusted for geriatric patients, (e.g., single room), is the evidence that modern ICU gravitate to more “patient friendly” design. Enhanced recovery programs are sometimes perceived by health management as more expensive in terms of environmental and design alterations. Based on available data from the United States, ERAS programs lead to reductions in lengths of hospital stay that range from 0.7 to 2.7 days and substantial direct cost savings (39).

## Conclusions and future directions

The Society of Critical Care Medicine's ICU Liberation initiative aims to liberate patients from the harmful effects of pain, agitation, and delirium in the ICU. The Society of Critical Care Medicine introduced the concept of ABCDEF bundle for intensive care: **A**ssess, Prevent, and Manage Pain, **B**oth Spontaneous Awakening Trials and Spontaneous Breathing Trials, **C**hoice of Sedation, **D**elirium: Assess, Prevent and Manage **E**arly Mobility and Exercise, **F**amily Engagement and Empowerment. This concept and detailed care bundles can be found on the website www.iculiberation.org for details.

Future directions for the improvement and enhancement of the patient recovery in SICU is in development and implementation of specific care bundles (are being) carried out by SICU multidisciplinary teams. We can utilize existing ones or prepare and modify local ones.

Quality guidelines do already exist (8, 12, 19, 20, 33), therefore implementation in everyday practice is the key for impact and enhancement of the patients recovery in SICU. More emphasis on pain treatment, as well as treatment of delirium and agitation, early nutritional screening and interventions, early mobilization of critically ill patients, family member involvement and paradigm shift in our mentality as an ICU team members, is necessary for change to happen.

Summary recommendations for ERAS in SICU are in Table 1.

**Table 1:** Summary of recommendations for ERAS in SICU.

1.Pain agitation delirium	1.a Pain treatment is precondition for other treatment options 1.b Implement existing protocols for sedation and analgesia in the SICU (or develop local ones) 1.c Implement tools for assessing the patient level of sedation and pain. 1.d Implement prevention and treatment strategies for postoperative delirium. 1.e Development and implementation of CARE BUNDLES for sedation, analgesia and postoperative delirium in each SICU. **Guidelines**: Clinical practice guidelines for the management of pain, agitation, and delirium in adult patients in the intensive care unit. *Crit Care Med* (2013) 41:263–306.
2.Nutrition	2.a. Care bundles for early nutritional interventions and implementation of nutritional protocols should be developed by multidisciplinary ICU teams. **Guidelines:** 2016 Guidelines for the Provision and Assessment of Nutrition Support Therapy in the Adult Critically Ill Patient. *JPEN* (2016) 40(2): 159 −211.
3.Early mobilization	3.a ERAS protocols encourage early mobilization of the patients. 3.b Early mobilization is possible and should be implemented by special multidisciplinary ICU team. **Guidelines:** Physiotherapy for adult patients with critical illness: recommendations of the European Respiratory Society and European Society of Intensive Care Medicine Task Force on Physiotherapy for Critically Ill Patients. *Intensive Care Med* (2008) 34:1188–1199.
4.Logistic issues	4.a. All team members must be familiar with protocols in order to implement them in their field of expertise. 4.b Implementation will require an organizational modifications in the preoperative anesthesia visit, preoperative preparation, intraoperative pain management or fluid management, modifications in postoperative care **Guidelines**:www.erassociety.org

## Limitatations of the review

One of the main limitations of this study is that these are recommendations for ERAS protocols in other settings, but there is not scientific data yet to show if they can be translated effectively into another (SICU) setting. This idea of incorporating ERAS protocols in SICU setting have great potential for future scientific research.

Second limitation pertains to the data we already have and that are proposed here to be used in SICU, such as existing protocols for assessment or management of sedation, analgesia, delirium, nutrition and early mobilization. Each and every item mentioned here is subject to some criticism in the present literature. For the present time, while waiting for further scientific refinement, those guidelines are the best scientific knowledge that we have. By using them we give structure and solid scientific background to our everyday work in SICU and their implementation can lead to optimization of various aspects of SICU practices.

## Author contributions

GJ: conceptualization, investigation, literature review, visualization, and made a final version of manuscript, language supervision. DJ: conceptualization, literature review, writing the manuscript. ML-S: conceptualization, literature review, writing the manuscript, supervision.

### Conflict of interest statement

The authors declare that the research was conducted in the absence of any commercial or financial relationships that could be construed as a potential conflict of interest. The reviewer DM and handling editor declared their shared affiliation.

## Abbreviations

RAS: Enhanced Recovery After surgery
ICU: intensive care unit
SICU: surgical intensive care unit
e CASH: early Comfort using Analgesian minimal Sedatives and maximal Human care
ICU PAD: intensive care unit pain agitation delirium
ESPEN: European Society for Enteral and Parenteral Nutrition
A.S.P.E.N: American Society for Enteral and Parenteral Nutrition
ESICM: European Society of Intensive Care Medicine
BW: body weight
ICUAW: intensive care unit-acquired weakness
ABCDEF bundle: Assess, Prevent, and Manage Pain, Both Spontaneous Awakening Trials and Spontaneous Breathing Trials, Choice of Sedation, Delirium: Assess, Prevent and Manage Early Mobility and Exercise, Family Engagement and Empowerment.

## References

[1] Kehlet H. Multimodal approach to control postoperative pathophysiology and rehabilitation. Br J Anaesth. (1997) 78:606–17. 9175983 10.1093/bja/78.5.606

[2] Lassen K Coolsen M Slim K Carli F Aguilar-Nascimento E Schäfer M. Pancreaticoduodenectomy: ERAS recommendations. Clin Nutr. (2013) 32:870–1. 10.1016/j.clnu.2012.08.011 23731515

[3] Steenhagen E. Enhanced recovery after surgery: it's time to change practice! Nutr Clin Pract. (2016) 31:18–29. 10.1177/0884533615622640 26703956

[4] Cannesson M Ani F Mythen M Kain Z. Anaesthesiology and perioperative medicine around the world: different names, same goals. Br J Anaesth. (2015) 114:8–9. 10.1093/bja/aeu265 25145355

[5] Vincent JL Hall JB Slutsky AS. Ten big mistakes in intensive care medicine. Intensive Care Med. (2014) 41:505–7. 10.1007/s00134-014-3570-7 25502093

[6] Vincent JL Shehabi Y Walsh TS Pandharipande PP Ball JA Spronk P Comfort and patient-centered care without excessive sedation: the eCASH concept. Intensive Care Med. (2016) 42:962–71. 10.1007/s00134-016-4297-4.7 27075762 PMC4846689

[7] Vincent JL Optimizing sedation in the ICU: the eCASH concept. Signa Vitae (2017) 13:10–13. 10.22514/SV133.062017.1

[8] Barr J Fraser GL Puntillo K Ely EW Gélinas C Dasta JF. Clinical practice guidelines for the management of pain, agitation, and delirium in adult patients in the intensive care unit. Crit Care Med. (2013) 41:263–306. 10.1097/CCM.0b013e3182783b72 23269131

[9] Chou R Gordon DB de Leon-Casasola OA Rosenberg JM Bickler S5 Brennan T. Management of Postoperative Pain: A Clinical Practice Guideline From the American Pain Society, the American Society of Regional Anesthesia and Pain Medicine, and the American Society of Anesthesiologists' Committee on Regional Anesthesia, Executive Committee, and Administrative Council. J Pain (2016) 17:131–57. 10.1016/j.jpain.2015.12.008 26827847

[10] Kotfis K Zegan-Baranska M Szydłowski L Zukowski M Ely E. Methods of pain assessment in adult intensive care unit patients — Polish version of the CPOT (Critical Care Pain Observation Tool) and BPS (Behavioral Pain Scale). Anaesthesiol Intensive Ther. (2017) 49:66–72. 10.5603/AIT.2017.0010 28362033

[11] Grover S Sarkar S Yaddanapudi LN Ghosh A Desouza A Basu D. Intensive Care Unit delirium: A wide gap between actual prevalence and psychiatric referral. J Anaesthesiol Clin Pharmacol. (2017) 33:480–6. 10.4103/0970-9185.222505 29416240 PMC5791261

[12] Baron R Binder A Biniek R Braune S Buerkle H Dall P. Evidence and consensus based guideline for the management of delirium, analgesia, and sedation in intensive care medicine. Revision 2015 (DAS-Guideline 2015) –short version. Ger Med Sci. (2015) 13:Doc19. 10.3205/000223 26609286 PMC4645746

[13] Ely E Gautam S Margolin R Francis J May L Speroff T. The impact of delirium in the intensive care unit on hospital length of stay. Intensive Care Med. (2001) 27:1892–900. 10.1007/s00134-001-1132-2 11797025 PMC7095464

[14] Mc Cusker J Cole MG Dendukuri N Belzile E Does delirium increase hospital stay? J Am Geriatr Soc. (2003) 51:539–46. 14687382 10.1046/j.1532-5415.2003.51509.x

[15] Wassenaar A Rood P Schoonhoven L Teerenstra S Zegers M Pickkers P. The impact of nUrsiNg DEliRium Preventive INnterventions in the Intensive Care Unit (UNDERPIN-ICU): a study protocol for a multi-centre, stepped wedge randomized controlled trial. Int J Nurs Stud. (2017) 68:1539–46. 10.1016/j.ijnurstu.2016.11.018 28013104

[16] Mitchell ML Kean S Rattray JE Hull AM Davis C Murfield JE Family intervention to reduce delirium in hospitalized ICU patients: A feasibility randomized controlled trial. Int Crit Care Nurs. (2017) 40:77–84. 10.1016/j.iccn.2017.01.001 28254205

[17] Kehlet H. The stress response to surgery: release mechanisms and the modifying effect of pain relief. Acta Chir Scand. (1988) 550(Suppl):22–8. 2652970

[18] Kehlet H. Surgical stress: the role of pain and analgesia. Br J Anaesth. (1989) 63:189–95. 2669908 10.1093/bja/63.2.189

[19] McClave SA Taylor BE Martindale RG Warren MM Johnson DR Braunschweig C 2016 Guidelines for the Provision and Assessment of Nutrition Support Therapy in the Adult Critically Ill Patient. J Parenter Enteral Nutr. (2016) 40:159–211. 10.1177/0148607115621863 26773077

[20] Kreymann KG Berger MM Deutz NE Hiesmayr M Jolliet P Kazandjiev G Nitenberg G. ESPEN Guidelines on enteral nutrition: intensive care. Clin Nutr. (2006) 25:210–23. 10.1016/j.clnu.2006.01.021 16697087

[21] Reintam BA Starkopf J Alhazzani W Berger MM Casaer MP Deane AM Early enteral nutrition in critically ill patients: ESICM clinical practice guidelines. Intensive Care Med. (2017) 43:380–98. 10.1007/s00134-016-4665-0 28168570 PMC5323492

[22] Wischmeyer PE. Tailoring nutrition therapy to illness and recovery. Crit Care (2017) 21(Suppl. 3):316. 10.1186/s13054-017-1906-8 29297385 PMC5751603

[23] Hoffer LJ Bistrian BR. What is the best nutritional support for critically ill patients? Hepatobiliary Surg Nutr. (2014) 3:172–4. 10.3978/j.issn.2304-3881.2014.08.03 25202692 PMC4141292

[24] Hoffer LJ Bistrian BR. Appropriate protein provision in critical illness: a systematic and narrative review. Am J Clin Nutr. (2012) 96:591–600. 10.3945/ajcn.111.032078 22811443

[25] Pavy-Le Traon A Heer M Narici MV Rittweger J Vernikos J. From space to Earth: advances in human physiology from 20 years of bed rest studies (1986–2006). Eur J Appl Physiol. (2007) 101:143–94. 10.1007/s00421-007-0474-z 17661073

[26] Dock W The evil sequelae of complete bed rest. JAMA (1944) 125:1083–85. 10.1001/jama.1944.02850340009004

[27] Nordon-Craft A Moss M Quan D Schenkman M. Intensive care unit-acquired weakness: implications for physical therapist management. Phys Ther. (2012) 92:1494–506. 10.2522/ptj.20110117 22282769 PMC3513482

[28] Stevens RD Dowdy DW Michaels RK Mendez-Tellez PA Pronovost PJ Needham DM. Neuromuscular dysfunction acquired in critical illness: A systematic review. Intensive Care Med. (2007) 33:1876–91. 10.1007/s00134-007-0772-2 17639340

[29] Latronico N Bolton CF. Critical illness polyneuropathy and myopathy: a major cause of muscle weakness and paralysis. Lancet Neurol. (2011) 10:931–41. 10.1016/S1474-4422(11)70178-8 21939902

[30] Hassan A Rajamani A Fitzsimons F The 'MOVIN' project (Mobilization Of Ventilated Intensive care patients at Nepean): A quality improvement project based on the principles of knowledge translation to promote nurse-led mobilization of critically ill ventilated patients. Int Critl Care Nurs. (2017) 42:36–43. 10.1016/j.iccn.2017.04.011 28552258

[31] Li Z Peng X Zhu B Zhang Y Xi X. Active mobilization for mechanically ventilated patients: A systematic review. Arch Phys Med Rehabil. (2013) 94:551–61. 10.1016/j.apmr.2012.10.023 23127305

[32] Schweickert WD Kress JP. Implementing early mobilization interventions in mechanically ventilated patients in the ICU. Chest (2011) 140:1612–7. 10.1378/chest.10-2829 22147819

[33] Gosselink R Bott J Johnson M Dean E Nava S Norrenberg M. Physiotherapy for adult patients with critical illness: recommendations of the European Respiratory Society and European Society of Intensive Care Medicine Task Force on Physiotherapy for Critically Ill Patients. Intensive Care Med. (2008) 34:1188–99. 10.1007/s00134-008-1026-7 18283429

[34] Adler J Malone D. Early mobilization in the intensive care unit: A systematic review. Cardiopulm Phys Ther J. (2012) 23:5–13. 22807649 PMC3286494

[35] Kehlet H Delaney CP Hill AG. Perioperative medicine – the second round will need a change of tactics. Br J Anaesth. (2015) 115:13–4. 10.1093/bja/aev098 25908052

[36] Barber EA Everard T Holland AE Tipping C Bradley SJ Hodgson CL Barriers and facilitators to early mobilization in intensive care: A qualitative study. Austr Crit Care (2015) 28:177–82. 10.1016/j.aucc.2014.11.001 25533868

[37] Kehlet H Fast-track surgery: A multidisciplinary collaboration. ICU Manage Pract. (2017) 17:138–9. 10.1302/2058-5241.2.160060

[38] Needham D Feldman D Kho M. The functional costs of ICU survivorship. Collaborating to improve post–ICU disability. Am J Respir Crit Care Med. (2011) 183:962–4. 10.1164/rccm.201012-2042ED 21498817

[39] Stone B Grant C Roda P Hobson D Pawlik T Wu L Wick E. Implementation costs of an enhanced recovery after surgery program in the United States: A financial model and sensitivity analysis based on experiences at a quaternary academic medical center. J Am Coll Surg. (2016) 222:219–25. 10.1016/j.jamcollsurg.2015.11.021 26774492

